# Meningomyeloencephalitis secondary to *Mycobacterium haemophilum* infection in AIDS

**DOI:** 10.1186/s40478-020-00937-2

**Published:** 2020-05-19

**Authors:** Sandra Leskinen, Xena Flowers, Katharina Thoene, Anne-Catrin Uhlemann, James E. Goldman, Richard A. Hickman

**Affiliations:** 1 Department of Pathology & Cell Biology, Columbia University Irving Medical Center, New York, NY 10032 USA; 2 Department of Medicine, Columbia University Irving Medical Center, New York, NY 10032 USA; 3 Microbiome & Pathogen Genomics Core, Columbia University Irving Medical Center, New York, NY 10032 USA

**Keywords:** *Mycobacterium haemophilum*, Non-tuberculous mycobacteria, HIV, AIDS, Rhomboencephalitis, Meningomyeloencephalitis

## Abstract

Infections by opportunistic non-tuberculous mycobacteria (NTM) are rising in global incidence. One emerging, slowly growing NTM is *Mycobacterium haemophilum,* which can cause skin, lung, bone, and soft tissue infections in immunocompromised patients as well as lymphadenitis in immunocompetent individuals. Detection of this microorganism is difficult using conventional culture-based methods and few reports have documented involvement of this pathogen within the central nervous system (CNS).

We describe the neuropathologic autopsy findings of a 39-year-old man with AIDS who died secondary to *M. haemophilum* CNS infection. He initially presented with repeated bouts of pyrexia, nausea and vomiting, and altered mental status that required numerous hospitalizations. CSF infectious workups were consistently negative. His most recent admission identified hyperintensities within the brainstem by MRI and despite antibiotic therapies for suspected CNS infection, he died. Autopsy revealed a swollen brain with marked widening of the brainstem. Microscopic examination of the brain and spinal cord showed focal lymphohistiocytic infiltrates, gliosis and neuronal loss that were associated with acid-fast bacilli (AFB). The brainstem was the most severely damaged and AFB were found to congregate along arterial territories lending support to the notion of hematogenous spread as a mechanism for the organisms’ dissemination. 16S rRNA sequencing on formalin-fixed paraffin-embedded tissue enabled post-mortem identification of *M. haemophilum*. This sequencing methodology may permit diagnosis on CSF intra-vitam.

## Introduction

Non-tuberculous mycobacterial (NTM) infections are generally seen in patients with impaired cell-mediated immunity but can also be found in patients without underlying disease [1]. Included in this category of NTM is *Mycobacterium haemophilum*, which is a slow-growing, acid-fast bacillus that is recognized to cause cutaneous, pulmonary, bone, and joint infections [2, 3]. This bacterium can infect immunocompromised patients and transplant recipients but may also affect immunocompetent individuals as a cause for cervical lymphadenitis (scrofula) [4–6]. *M. haemophilum* infection within the central nervous system (CNS) is rare and, to our knowledge, only six reports in the English literature have described the neuropathologic findings of the infection, all of which are based on surgical biopsy material [7–13]. This underreporting may relate to the inherent challenges of culturing this organism using conventional microbiological methods [3]. We describe the post-mortem neuropathologic findings of an AIDS patient with meningomyeloencephalitis as a result of *M. haemophilum* infection. Speciation of the microorganism was only rendered after death by using 16S rRNA sequencing on the formalin-fixed, paraffin-embedded tissue. The histopathology and pattern of distribution of the microorganism are discussed.

## Case presentation

**Fig. 1:**
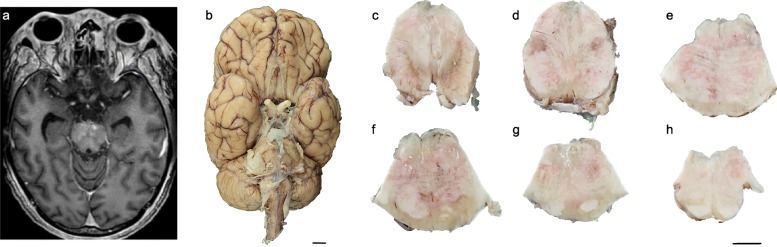
Radiologic and macroscopic autopsy findings of this patient. **a** T1 FLAIR weighted MRI showing multiple hyperintensities within the upper pons. **b** Inferior aspect of the brain demonstrating a markedly widened brainstem. **c-h** Transverse sections of the brainstem from rostral to caudal commencing from the caudal midbrain to the rostral medulla oblongata. Scale bars: **b**, 1 cm; **c-h**, 1 cm. **The high resolution source of Figure 1 is available as Additional file**1

The decedent was a 39-year-old man with newly diagnosed HIV/AIDS, who had recurrent bouts of pyrexia of unknown origin, nausea and vomiting, and altered mental status that required repeated admissions at an outside hospital over a four-month period. Aside from the recent diagnosis of HIV/AIDS, there was no other significant past medical history. Assessment of his respiratory system showed no abnormalities and chest radiographs showed no focal consolidation, atelectasis, or pleural fluid accumulation. Microbiological analysis of cerebrospinal fluid was negative for infectious organisms. On his last admission, cranial imaging identified a brainstem mass and the patient was transferred to our neurological intensive care unit requiring intubation for suspected aspiration pneumonia. At the time of transfer, his CD4 cell count was 3 cells/mm^3^ with a viral load of 46,000 copies/mL and he was initiated on antiretroviral therapy (ART). He underwent MRI which showed a new nodular brainstem enhancement and residual leptomeningeal enhancement (Fig. 1a) in addition to a 7 mm enhancing region at the cervicomedullary junction in the anterior cervical spinal cord. Analysis of his cerebrospinal fluid (CSF) at this time showed an elevated protein (97 mg/dL; normal: 15–45 mg/dL) and glucose (80 mg/dL; normal: 40–70 mg/dL) with a lymphocytosis (100 cells/mm^3^). Quantitative PCR was negative for JC virus and a meningitis/encephalitis PCR panel was negative for *Escherichia coli*, *Haemophilus influenzae*, *Listeria monocytogenes*, *Neisseria meningitidis*, *Streptococcus pneumoniae*, Cytomegalovirus, Enterovirus, Human Herpes Virus-6, Herpes Simplex Virus-1, Herpes Simplex Virus-2, Human Parechovirus, Varicella Zoster Virus, and *Cryptococcus neoformans*. Furthermore, culture of CSF, including cultures for acid-fast bacilli (AFB), showed no growth of microorganisms. Despite broad-spectrum antibiotics for suspected rhomboencephalitis, he died. Other than his known HIV status, at no point during his hospitalizations was an infectious agent identified.

An autopsy restricted to the brain and spinal cord was performed with written informed consent obtained from the family. The fresh brain weight was increased (1525 g) and the leptomeninges were thickened multifocally, being most pronounced over the midbrain, pons, and right middle cerebellar peduncle. The brainstem was diffusely widened (Fig. 1b) and the cerebral gyri were swollen. Coronal sections of the cerebral hemispheres revealed marked, bilateral dilatation of the lateral and third ventricles. Transverse sections of the brainstem showed poor demarcation of the gray and white matter (Fig. 1c-h). The substantia nigra was pale and the cerebral aqueduct was obliterated (Fig. 1c). The pons and medulla oblongata appeared mottled and hyperemic with white patches over the basis pontis (Fig. 1d-h).

Microscopically, leptomeningeal lymphohistiocytic infiltrates were seen multifocally. Widespread damage was seen in the brain and spinal cord with focal lymphohistiocytic infiltrates, neuropil vacuolation, neuronal loss, gliosis, and perivascular inflammation. This was found throughout the examined neocortex with relative sparing of the corpus striatum and thalamus. The subcortical white matter showed patches of myelin pallor and were also associated with lymphohistiocytic inflammation.

**Fig. 2:**
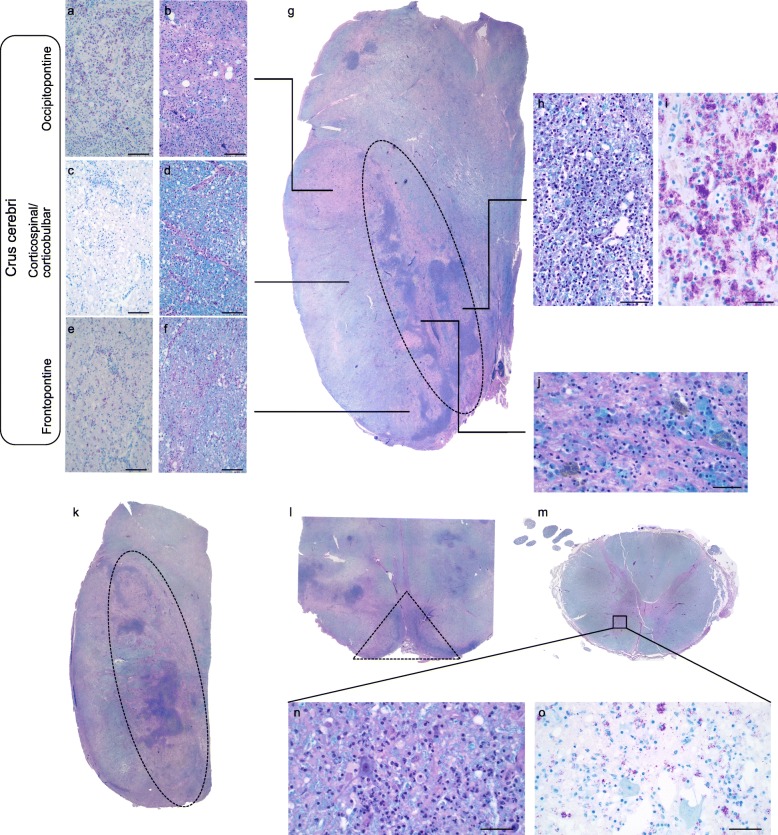
Microscopic findings of this patient. **a-b** Representative micrograph of the AFB and LH&E of the occipitopontine tract. **c-d** Representative micrographs of the AFB and LH&E of the corticospinal/ corticobulbar tract. **e-f** Representative micrographs of the AFB and LH&E of the frontopontine tract. **g** Whole section image of the left half midbrain at the level of the superior cerebellar peduncle. **h-i** Representative image of the most severely inflamed region in the midbrain highlighting extensive lymphohistiocytic infiltrate and abundant AFB within macrophages. **j** Representative image showing macrophages containing myelin and neuromelanin in the substantia nigra. **k** Whole section image of the left upper pons. **l** Micrograph of the ventral rostral medulla. **m** Whole section image of thoracic cord. **n** LH&E stain highlights ventral horn neuron surrounded by lymphohistiocytic inflammation. **o** Macrophages containing AFB surround apparently normal ventral horn neurons. Area outlined by dashed lines indicate arterial territories supplying respective brainstem levels **(g, k, l)**. Scale bars: **a-f, h**: 100 μm, **i-j, n-o**: 50 μm. **The high resolution source of Figure 2 is available as Additional file**1

The most severe damage in the CNS involved the brainstem. The degree of injury in the brainstem was not diffuse but varied throughout. In the midbrain, at the level of the decussation of the superior cerebellar peduncles (Fig. 2g), the most dramatic inflammatory infiltrate was in the midline and extended laterally to involve the substantia nigra (Fig. 2g-h). The substantia nigra showed marked neuronal loss and was associated with neuromelanin-laden macrophages (Fig. 2k). Gram, periodic acid-Schiff (PAS) and Grocott’s methenamine silver (GMS) stains were negative for organisms. AFB stains revealed numerous rod-shaped microorganisms within epithelioid histiocytes, thus compatible with NTM infection. The severely damaged midbrain harbored the highest density of AFB (Fig. 2i).

To determine the species of the AFB, DNA was extracted from formalin-fixed, paraffin-embedded material of the midbrain (ReliaPrep FFPE gDNA Miniprep System, Zymo). Using primers targeting the 16S V3-V4 rRNA region, DNA was amplified and Sanger sequencing was performed. Using BLAST searching on NCBI, the sequence matched closest to a group of non-tuberculous mycobacterial references sequences, including *M. haemophilum* and *M. riyadhense* at 99% identity. Therefore, the mycobacterial hsp65 protein gene region was amplified with the forward primer (5′ ACCAACGATGGTGTGTCCAT 3′) and the reverse primer (5′ CTTGTCGAACCGCATACCCT 3′) [14]. The obtained hsp65 sequence ranked highest with 99.72% identity to *M. haemophilum*.

## Discussion and conclusion

Several histologic findings provide clues into the pathophysiology of this infection. Firstly, the density of AFB correlated with the degree of parenchymal damage. This gradient of injury and AFB density was evident in the crus cerebri. The most severe myelin loss was seen in the occipitopontine tract, which had the greatest density of AFB, whereas fewer AFB were present in the more preserved corticospinal tract (Fig. 2a-f). Secondly, the distribution of AFB followed arterial territories. At the level of the midbrain and pons, the density of AFB was highest along the territory of the paramedian branch of the basilar artery while at the rostral medulla oblongata, it followed the anterior spinal artery (Dashed outline of Fig. 2g, k, l). Chronic inflammation was also seen within the anterior horns of the spinal cord, which is also a territory of the anterior spinal artery (Fig. 2n-o) and showed formation of microglial nodules and abundant AFB.

The arterial distribution of damage supports the concept of hematogenous seeding from a non-CNS source. NTM are thought to reach the CNS via hematogenous spread from the gastrointestinal tract or the lungs [15]. The autopsy was CNS-restricted and so unfortunately identification of an extra-CNS origin could not be ascertained. In immunocompromised patients, *M. haemophilum* most commonly infects the skin, while pulmonary, bone, and joint infections are less frequent systems of involvement [6, 16]. Aside from concerns of an aspiration pneumonia later in the patient’s course, there were no pulmonary, dermatologic, or joint complaints documented during his clinical course. However, the possibility remains that a focal, cutaneous infection may have gone undetected. It is also uncertain as to the source of infection for this patient since little is known of the environmental habitat in which *M. haemophilum* dwells. Potential sources of *M. haemophilum* infection include drinking water, contact with certain animals, as well as exposure to cosmetics [2, 7, 17, 18].

In contrast to one prior surgical report of a single granulomatous mass secondary to *M. haemophilum*, the meningomyeloencephalitis did not show evidence of granulomata, emphasizing that this disease can have variation in clinical presentations and histopathologic findings [7]. Conventional culture methods that apply for other mycobacteria do not generally succeed with *M. haemophilum*. Identification of this fastidious organism requires a strong index of suspicion, needing iron-supplemented media and culturing at cooler temperatures (30–32 °C) [19]. These necessary temperatures for optimal growth have been suggested as to why the organism favors infection in distal limb sites [20]. 16S rRNA sequencing is another method of identification and was critical in this challenging post-mortem diagnosis. In circumstances of suspected culture-negative meningoencephalitis, 16S rRNA sequencing of CSF may be another useful diagnostic tool in accurately identifying *M. haemophilum* for better informed clinical care [21, 22].

## Supplementary information

**Additional file 1.** High resolution source for Figures 1 & 2.

## Data Availability

N/A.

## Abbreviations

AFB: Acid fast bacillus
ART: Anti-retroviral therapy
CNS: Central Nervous System
CSF: Cerebrospinal fluid
GMS: Grocott’s methenamine silver
LH&E: Luxol fast blue counterstained with hematoxylin and eosin
NTM: Non-tuberculous mycobacterium
PAS: Periodic acid-Schiff and (GMS)
