# Supplementary figures and images for: Meningomyeloencephalitis secondary to Mycobacterium haemophilum infection in AIDS
Source: Acta Neuropathol Commun. 2020 May 19;8:73. doi: 10.1186/s40478-020-00937-2 (PMC7236527; doi:10.1186/s40478-020-00937-2)

Crus cerebri

Corticospinal/  
corticobulbar

Frontopontine

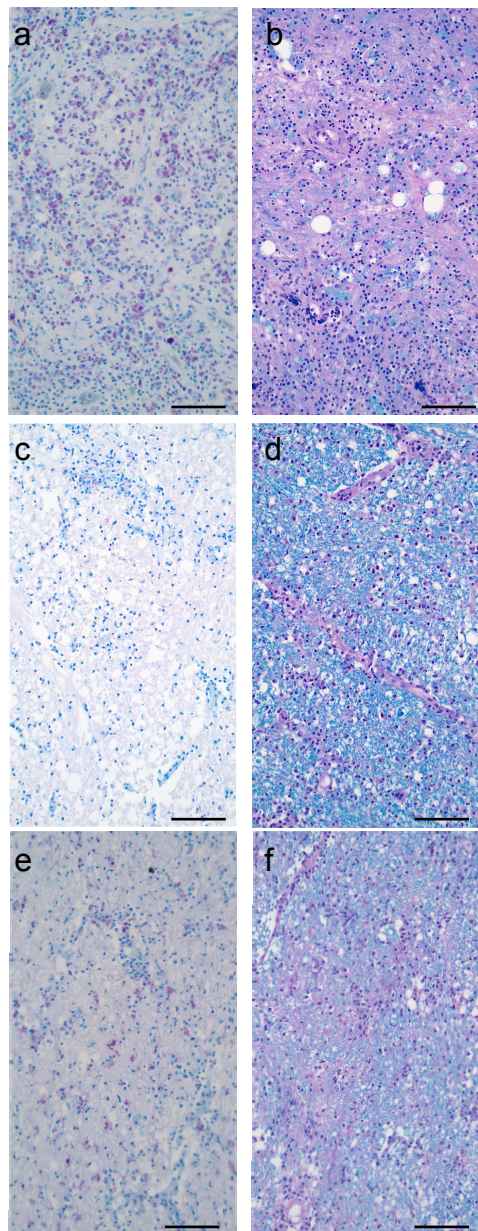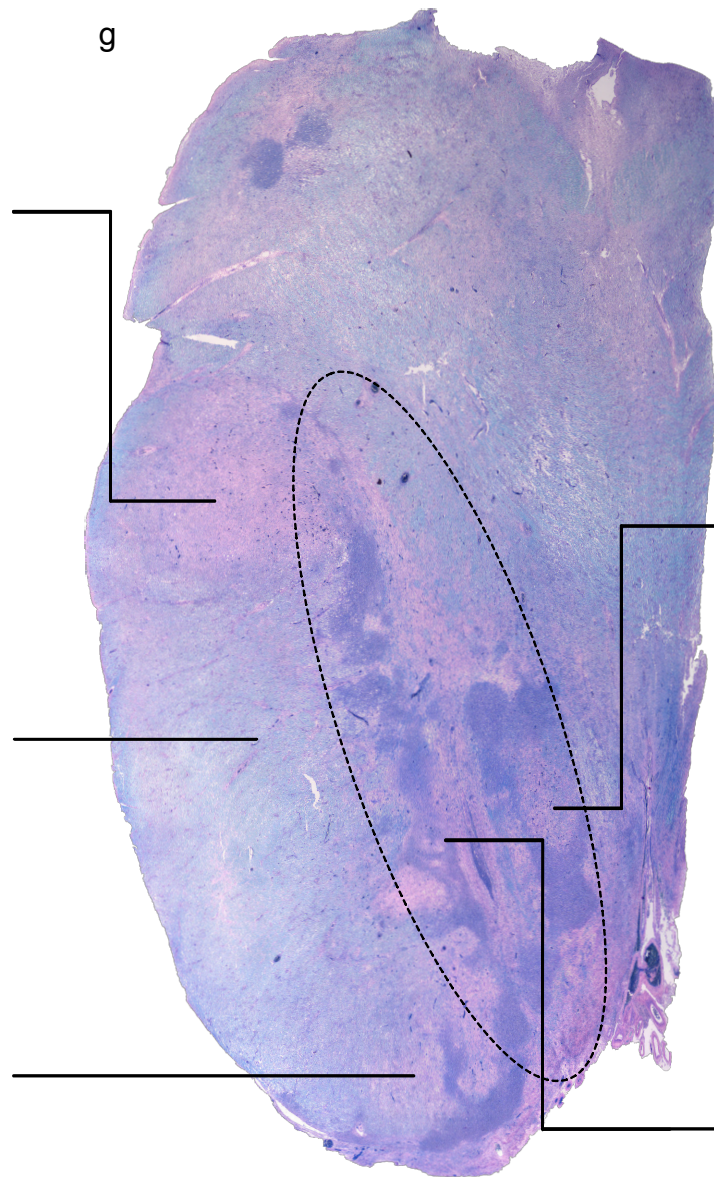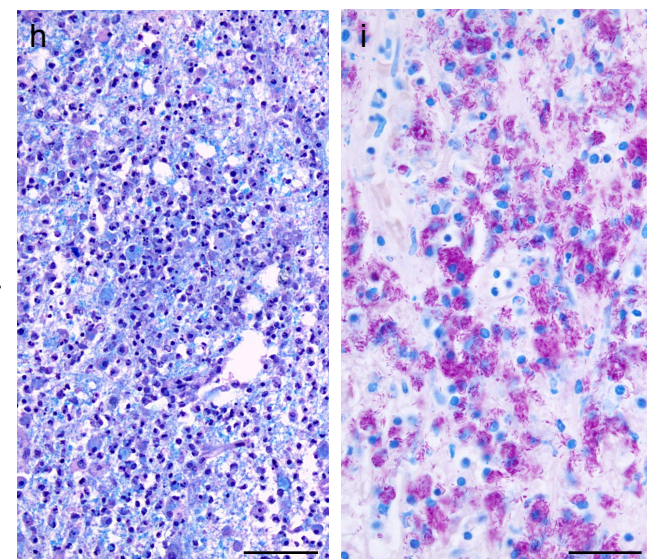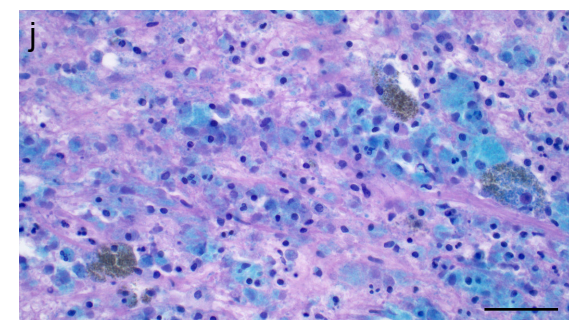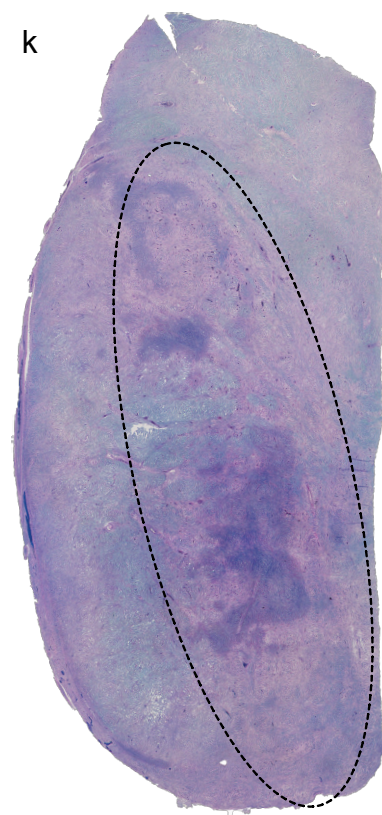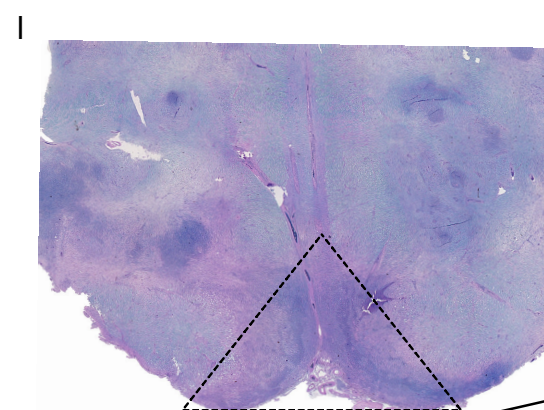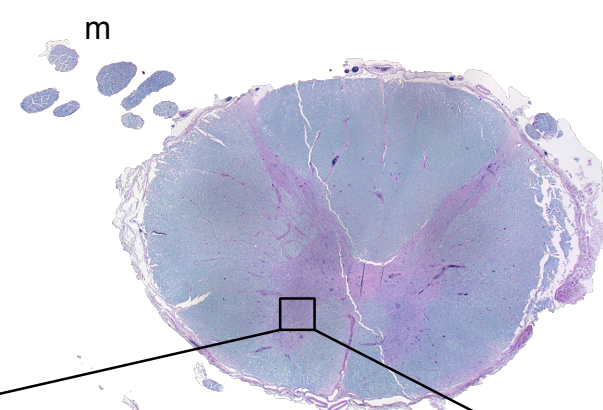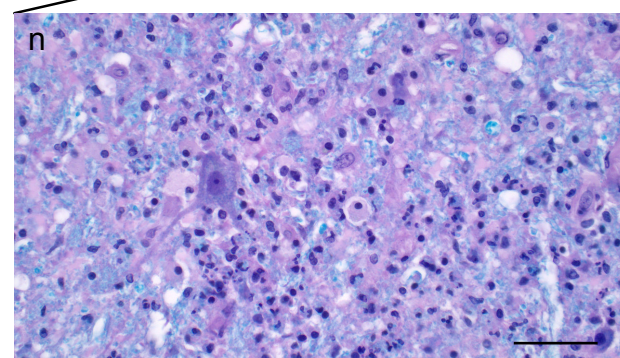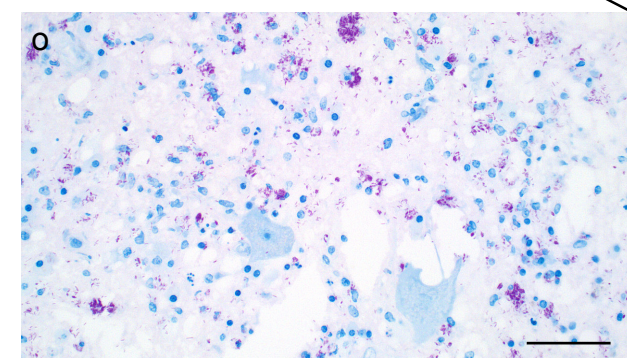

Supplement: Supplementary file 1 — Additional file 1. High resolution source for Figures 1 & 2. [file 40478_2020_937_MOESM1_ESM.zip › Figure 2 - Leskinen et. al..pdf]

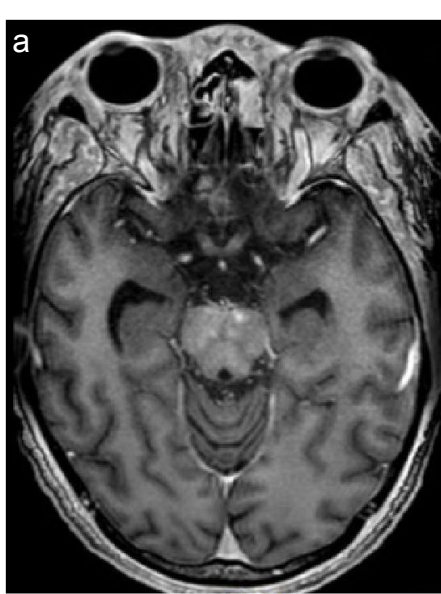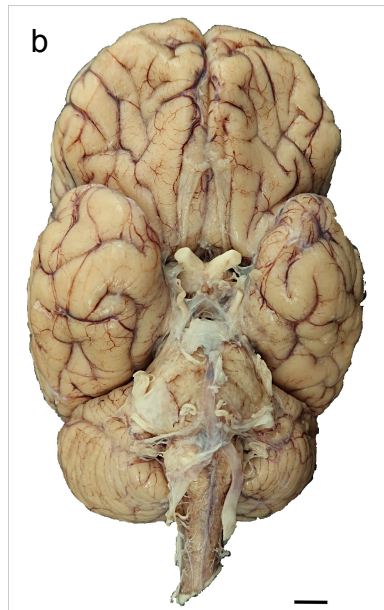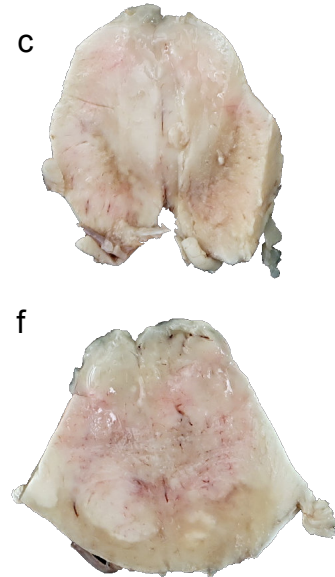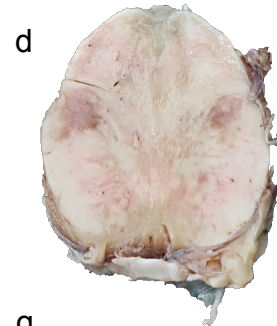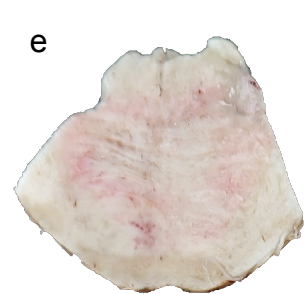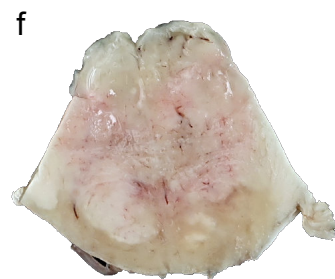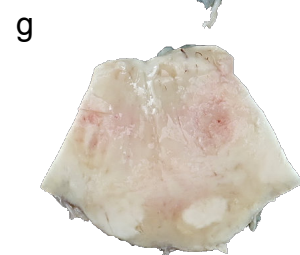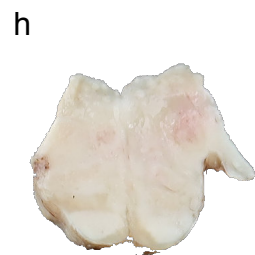

Supplement: Supplementary file 1 — Additional file 1. High resolution source for Figures 1 & 2. [file 40478_2020_937_MOESM1_ESM.zip › Figure 1 - Leskinen et. al.pdf]
